# Real-World Cardiovascular Research Using the German IQVIA Disease Analyzer Database: Methods, Evidence, and Limitations (2000–2025)

**DOI:** 10.3390/jcdd13020061

**Published:** 2026-01-24

**Authors:** Karel Kostev, Marcel Konrad, Mark Luedde

**Affiliations:** 1 Marburg University, University Hospital, 35043 Marburg, Germany; 2 Epidemiology, IQVIA, 60549 Frankfurt am Main, Germany; 3 Health & Social, FOM University of Applied Sciences for Economics and Management, 60486 Frankfurt am Main, Germany; 4 Medical Clinic I, Cardiology and Angiology, University Hospital of Giessen and Marburg, Campus Giessen, 35392 Giessen, Germany; 5 Department of Cardiology, Christian-Albrechts-University of Kiel, 24118 Kiel, Germany

**Keywords:** cardiovascular diseases, database, real-world evidence, real-world data, epidemiology

## Abstract

Cardiovascular diseases (CVDs) remain the leading cause of morbidity and mortality worldwide. This increases the demand for real-world evidence to complement findings from randomized controlled trials. The German IQVIA Disease Analyzer (DA) database, which is populated with anonymized electronic medical records from general practitioners and specialists, has become an increasingly valuable source for cardiovascular research. Over the past two decades, and especially between 2020 and 2025, numerous epidemiological studies have used this database to explore associations between cardiovascular risk factors, comorbidities, therapeutic patterns, and cardiovascular outcomes in large, broadly representative outpatient populations. This review synthesizes evidence from 13 selected DA-based studies examining atrial fibrillation, heart failure, cardiometabolic disease, lipid management, non-alcoholic fatty liver disease (NAFLD)–related cardiovascular risks, cerebrovascular complications, COVID-19-associated vascular events, and modifiable behavioral and anthropometric factors. These studies were selected based on predefined criteria including cardiovascular relevance, methodological rigor, large sample size, and representativeness of key disease domains across the 2000–2025 period. Eligible studies were identified through targeted searches of peer-reviewed literature using the German IQVIA Disease Analyzer database and were selected to reflect major cardiovascular disease domains, risk factors, and therapeutic areas. Across disease domains, the reviewed studies consistently demonstrate the DA database’s capacity to identify reproducible associations between cardiometabolic risk factors, comorbidities, and cardiovascular outcomes in routine outpatient care. While causal inference is not possible, the database enables the identification of clinically meaningful associations that inform hypothesis generation, help quantify disease burden, and highlight gaps in prevention or treatment. The database’s strengths include large sample sizes (often exceeding 100,000 patients), long follow-up periods, and high external validity, while limitations relate to coding accuracy, residual confounding, and the absence of detailed clinical measures. Collectively, the evidence underscores the importance of the DA database as a crucial platform for real-world cardiovascular research.

## 1. Introduction

Real-world data have become increasingly important for understanding disease patterns, treatment utilization, and outcomes under routine clinical conditions, and this is particularly true in cardiovascular epidemiology [1,2,3,4,5,6,7,8,9]. While randomized trials remain essential for assessing treatment efficacy under controlled conditions, their strict eligibility criteria and focus on internal validity can limit the generalizability of findings to broader, real-world patient populations [10,11,12,13]. By contrast, real-world data allow researchers to examine disease patterns, therapeutic trends, and associations between comorbidities and cardiovascular outcomes in everyday clinical practice [1,2,3,4,5]. The German IQVIA Disease Analyzer database is one of the most widely used outpatient routine care databases for this purpose. It contains longitudinal information on diagnoses, prescriptions, referrals, demographic characteristics, and clinical parameters recorded during routine practice. Its representativeness with respect to the German outpatient population has led to its use in hundreds of peer-reviewed studies, including a growing body of cardiovascular research.

Despite the increasing use of real-world databases in cardiovascular research, existing evidence derived from the Disease Analyzer database remains fragmented across individual disease-specific studies. To date, no comprehensive synthesis has integrated these findings across cardiovascular domains or examined their consistency, methodological patterns, and limitations over an extended time horizon. This gap limits the interpretability and contextualization of Disease Analyzer–based cardiovascular research.

In contrast to previous overviews of real-world cardiovascular databases that typically focus on single disease entities, specific therapies, or limited time frames, this review provides a longitudinal synthesis of cardiovascular research derived from the Disease Analyzer database over a 25-year period. By integrating evidence across multiple cardiovascular domains within a single nationally representative outpatient database, the review offers new insights into the consistency, evolution, and limitations of real-world cardiovascular associations observed in routine clinical practice.

Accordingly, an unmet need exists for an integrative synthesis that contextualizes Disease Analyzer–based cardiovascular findings across disease entities, methodological approaches, and time periods, while explicitly addressing strengths, limitations, and interpretative boundaries.

This review aims to synthesize the cardiovascular evidence derived from the DA database from 2000 to 2025, focusing on 13 rigorous studies. These studies examine a wide range of associations relevant to cardiovascular research, including atrial fibrillation (AF), heart failure (HF), lipid management, cardiometabolic risk factors, NAFLD, cerebrovascular events, COVID-19–related vascular outcomes, and associations between behavioral or psychiatric factors and cardiovascular complications (Table 1). By collecting and interpreting these findings, this review highlights the methodological value and clinical relevance of DA-based cardiovascular research.

## 2. Disease Analyzer Database

The studies included in this review were identified through targeted searches of peer-reviewed publications using the German IQVIA Disease Analyzer database published between 2000 and 2025. Inclusion criteria were: (1) a primary focus on cardiovascular outcomes or major cardiovascular risk factors; (2) use of retrospective cohort, case–control, or cross-sectional designs; (3) sufficiently large sample sizes to ensure statistical robustness; and (4) publication in international peer-reviewed journals. The 13 studies discussed do not represent the totality of cardiovascular research using the Disease Analyzer database but were selected to reflect key disease domains, methodological diversity, and clinical relevance. Accordingly, this work follows a structured narrative review approach rather than a formal systematic review. A systematic review framework was not applied because the included studies address diverse cardiovascular outcomes, exposures, and analytical approaches, which preclude meaningful quantitative synthesis or uniform risk-of-bias assessment.

The DA database obtains routine clinical data directly from the practice management systems used by general practitioners, diabetologists, cardiologists, neurologists, and other office-based specialists in Germany [14,15,16,17,18,19,20,21,22,23,24,25,26]. In contrast to German claims databases or hospital-based registries, the Disease Analyzer database captures longitudinal outpatient clinical data directly from physician documentation, including diagnoses, prescriptions, and selected clinical parameters. This structure enables detailed analyses of disease onset, treatment initiation, and comorbidity development in routine ambulatory care, which is not possible using administrative billing data alone.

Based on this database structure, the studies summarized in Table 1 were identified using predefined selection criteria. Eligible publications had to (1) be published between 2020 and 2025, (2) focus primarily on cardiovascular diseases or major cardiovascular risk factors, (3) use the German IQVIA Disease Analyzer database as the main data source, (4) be published in the English language, and (5) be indexed in PubMed. These criteria were applied to capture recent, methodologically comparable Disease Analyzer–based cardiovascular studies across major disease domains. The selected studies do not represent the totality of cardiovascular research conducted using the Disease Analyzer database, but rather a structured thematic sample intended to illustrate key applications, findings, and limitations of real-world cardiovascular research in German outpatient care.

Diagnoses are coded using the ICD-10 system, prescriptions follow the Anatomical Therapeutic Chemical (ATC) classification system, and laboratory and clinical measurements are documented where available. Practices are sampled to ensure national representativeness in terms of practice type, size, and regional distribution. Longitudinal patient records enable retrospective cohort, case–control, and cross-sectional analyses.

The DA database encompasses approximately 3% of all outpatient practices in Germany, including general practitioners, internists, diabetologists, cardiologists, neurologists, and other office-based specialists. Practices are selected using a stratified sampling procedure to ensure representativeness with respect to regional distribution, practice type, and patient volume. The database contains anonymized electronic medical records for more than 15 million individuals since the early 2000s. Because data are generated during routine clinical care rather than through active recruitment, all documented patients form part of the study population. Individual studies define their own observation periods, typically ranging from 5 to 15 years, depending on exposure and outcome definitions. Cardiovascular studies frequently include cohorts of more than 100,000 patients, enabling robust epidemiologic analyses.

Studies included in this review typically use matching strategies such as propensity score matching or exact matching based on age, sex, index year, and comorbidities. Statistical methods often include Cox regression or logistic regression models to assess associations between exposures and outcomes. It is important to note that the database does not allow for causal inference because the possibility of confounding by unmeasured variables cannot be excluded. Nevertheless, the database’s large sample sizes and broad coverage of chronic diseases make it highly suitable for descriptive epidemiology and association research.

Although this review does not follow a formal systematic review protocol, study relevance was evaluated based on predefined criteria, including cardiovascular focus, sample size, analytical rigor (e.g., matching strategies and regression models), and clinical interpretability. The aim was to synthesize representative and methodologically sound evidence rather than to exhaustively catalog all published Disease Analyzer studies.

Across the 13 reviewed studies, retrospective cohort designs were most commonly applied [14,15,16,18,19,21,22,25,26], typically using Cox proportional hazards models to estimate incidence associations. Case–control studies [23,24] employed multivariable logistic regression. Several studies additionally used matching strategies—including exact matching and propensity score matching on variables such as age, sex, index year, and comorbidities—to enhance internal validity [14,15,16,18,22,23,24]. Follow-up durations ranged from one to ten years, depending on outcome frequency. These methodological features support the manuscript’s statements regarding analytical rigor, internal validity, and the longitudinal nature of DA-based cardiovascular research.

## 3. Evidence from Studies on Atrial Fibrillation

Atrial fibrillation is one of the most common cardiovascular disorders in Europe [27]. Several DA-based studies have examined AF as either an outcome or an exposure, highlighting the complex interplay between cardiometabolic, behavioral, and demographic risk factors [14,15]. One major study investigated sex-related differences in the initiation of antithrombotic therapy following an AF diagnosis. This study included 166,005 patients [14]. Women were older at diagnosis (76.2 vs. 73.2 years) and were significantly less likely to receive antithrombotic therapy within 30 days after AF diagnosis compared with men (13.2% vs. 15.5%). Stroke-risk scores were similar between sexes, indicating that differences in clinical risk do not fully explain the treatment disparity. These findings are consistent with previous research showing that female AF patients remain undertreated with respect to oral anticoagulation [28,29,30].

Another large DA-based study examined the association between overweight or obesity and incident AF using data from approximately 392,000–400,000 individuals [15]. During 10 years of follow-up, AF incidence increased progressively across BMI categories: normal weight 7.2%, overweight 10.1%, and obesity 13.2%. Obesity was associated with a substantially higher AF risk (hazard ratio 1.43, 95% CI 1.38–1.48), with somewhat stronger associations observed in men. These results align with previous population-based studies demonstrating a dose–response relationship between higher BMI and AF risk [31,32].

Taken together, these AF-related studies reveal consistent effect directions across sex-related and anthropometric exposures. Although the study designs differ, all analyses used large, representative outpatient cohorts and adjusted for demographic and clinical factors. Shared limitations include the potential for residual confounding due to missing information on lifestyle factors and the lack of detailed clinical parameters such as AF burden and echocardiographic findings.

## 4. Lipid Management and Cardiometabolic Risk

A DA-based study including 32,963 very high cardiovascular-risk patients quantified their “distance to LDL-C goal,” defined as the percentage LDL-C reduction required to reach the thresholds set by the 2016 and 2019 ESC/EAS guidelines [19]. The majority of patients required substantial additional LDL-C lowering to achieve guideline-recommended targets, indicating a considerable unmet need in the outpatient setting. The study showed that even among patients already receiving lipid-lowering therapy, LDL-C values frequently remained far above recommended levels, underscoring persistent treatment gaps in German routine care.

Another DA-based study evaluated whether adults with ADHD are more likely to receive a diagnosis of dyslipidemia [20]. The study population consisted of 32,202 adults. Adults with ADHD had higher rates of dyslipidemia than individuals without ADHD, suggesting that ADHD-related lifestyle patterns or psychotropic medication profiles may influence lipid metabolism and cardiometabolic health.

A third large study, including 657,310 outpatients, examined the association between body height and cardiovascular diseases [21]. Taller height was inversely associated with coronary heart disease, whereas the incidence of atrial fibrillation and venous thromboembolism increased with increasing height. This pattern is consistent with previous literature showing that height exhibits outcome-specific associations with cardiovascular risk [33,34,35].

Overall, the DA-based studies on lipid management and cardiometabolic risk highlight both classical risk factors (such as LDL-C levels) and non-traditional determinants such as ADHD or height. Because lipid values and lifestyle data are not consistently documented across outpatient practices, residual confounding and incomplete laboratory data may influence effect estimates. However, the large sample sizes and consistency with external epidemiological research support the validity of the observed associations.

## 5. Heart Failure and Related Comorbidities

One study, including 123,516 HF cases and 123,516 matched controls, examined the association between sleep disorders and incident HF [16]. Patients with previously diagnosed sleep disorders had a significantly higher risk of developing HF. Because sleep disorders such as insomnia or sleep-related breathing disturbances are often underdiagnosed in primary care, the observed associations likely underestimate the true effect.

Another study involving 173,966 individuals analyzed the relationship between non-alcoholic fatty liver disease (NAFLD) and new-onset HF [22]. NAFLD was associated with a markedly increased HF risk, with approximately a 1.5-fold elevation even after adjustment for cardiometabolic factors. Because NAFLD is undercoded in outpatient care, diagnosed patients often represent more advanced disease stages, which may amplify the observed effect.

Loosen et al. investigated antibiotic use in 162,188 individuals and found an association between prior antibiotic exposure and incident HF [23]. The relationship appeared stronger with increasing antibiotic use. Since repeated antibiotic prescriptions may indicate a higher burden of infection or systemic inflammation, confounding by underlying morbidity may partly explain this association.

A further DA-based study examined comorbidities at the time of first HF diagnosis in 324,492 patients [24]. Nineteen out of 37 chronic conditions were significantly more frequent in patients with HF than in matched controls, illustrating the extensive multimorbidity burden present at HF onset.

Another study evaluated HF incidence in patients with alcoholic and non-alcoholic liver cirrhosis [18]. After up to 10 years of follow-up, HF occurred in 20.9% of alcoholic cirrhosis patients versus 10.3% of controls and in 23.0% of non-alcoholic cirrhosis patients versus 14.2% of controls. The hazard ratios were 2.07 (95% CI 1.85–2.31) for alcoholic cirrhosis and 1.70 (95% CI 1.56–1.82) for non-alcoholic cirrhosis. These findings highlight the systemic cardiovascular effects of chronic liver disease.

Across all HF studies, the DA database consistently identifies increased HF risk across multiple exposures, including sleep disorders, NAFLD, cirrhosis, infection burden, and multimorbidity. Limitations include the potential underdiagnosis of HF in outpatient care, incomplete exposure documentation, and limited availability of echocardiographic or biomarker data. Despite these challenges, the overall pattern of results aligns with known HF pathophysiology.

## 6. Vascular and Cerebrovascular Disease

Several DA-based studies have addressed vascular and cerebrovascular conditions. A matched cohort analysis investigated the relationship between schizophrenia and cardiovascular diseases [17]. Schizophrenia was associated with a significantly higher risk of heart failure (HR 1.33, 95% CI 1.20–1.48) and a lower risk of atrial fibrillation or atrial flutter (HR 0.77, 95% CI 0.67–0.89). This finding aligns with the broader international literature that highlights the burden of psychiatric-cardiac comorbidity [36,37,38,39]. 

Another study assessed the relationship between glucose-lowering medications and incident stroke or transient ischemic attack in patients with type 2 diabetes [26]. The study compared stroke risks across different antidiabetic drug classes. Although the DA database offers large sample sizes and long follow-up, treatment-selection bias and residual confounding remain important considerations when interpreting differences between drug classes.

A final study including 58,904 patients compared vascular event risk after COVID-19 infection with that after respiratory tract infections (RTI) [25]. The study found no significantly increased risk of first-ever vascular events following COVID-19 when compared with RTI. By using RTI as a comparator, the authors reduced confounding related to healthcare-seeking behavior, although incomplete capture of hospital-based vascular events must be considered.

Together, these studies demonstrate that the DA database can be used to examine vascular risks associated with psychiatric illness, pharmacologic therapy, and infectious diseases. Limitations such as incomplete lifestyle documentation, underreporting of hospital events, and potential residual confounding need to be considered, but overall, the results are consistent with established epidemiological findings and illustrate the broad applicability of the database for vascular research.

## 7. Strengths and Limitations

The DA database has several key strengths that make it valuable for cardiovascular research. Its large sample sizes enable robust statistical analyses and help detect associations that would not be identifiable in smaller cohorts. Many of the studies included hundreds of thousands of patients. In the reviewed studies, sample sizes ranged from 32,202 individuals in the ADHD–dyslipidemia analysis [20] to 657,310 individuals in the height and cardiovascular disease study [21]. Most atrial fibrillation and heart failure studies included more than 120,000–170,000 participants [14,15,16,18,22,23,24]. These large cohorts provide sufficient statistical power to detect clinically meaningful associations, including for relatively infrequent cardiovascular outcomes. Such sample sizes provide high statistical power and increase the reliability of observed associations.

Another strength of the database lies in its representativeness. The DA database captures outpatient care across Germany, which includes general practitioners and specialists. This broad inclusion enhances external validity and ensures that findings reflect real clinical practice. Conditions such as diabetes, hypertension, AF, and hyperlipidemia are largely managed in outpatient settings, making the DA database especially suitable for cardiovascular studies.

The longitudinal structure of the data allows for the study of disease progression and incident outcomes, as demonstrated by investigations into HF development [16], AF incidence with respect to different risk factors [15], and stroke incidence under specific medications [26]. This longitudinal capability allows researchers to examine associations across years of follow-up and offers insights into temporal relationships critical for chronic disease epidemiology.

From a clinical perspective, several findings—such as the associations between obesity and atrial fibrillation or between NAFLD and heart failure—are consistent with existing evidence and may reinforce clinical awareness of established risk patterns. In contrast, associations involving antibiotic exposure or psychiatric disorders should primarily be interpreted as hypothesis-generating and warrant further investigation before clinical implications can be drawn.

Furthermore, the DA database allows for the examination of comorbidity clusters, as evidenced by studies linking NAFLD with HF [22], cirrhosis with HF [18], and psychiatric disorders with cardiovascular outcomes [17]. The ability to evaluate overlapping disease processes is particularly valuable in cardiovascular epidemiology, where multimorbidity is the rule rather than the exception.

Additionally, DA studies can rapidly generate real-world evidence in emerging situations. This was evident in the COVID-19 vascular event study [25], which provided early insights into cardiovascular risks following SARS-CoV-2 infection. This rapid generation of evidence can be essential for informing health systems and clinicians under evolving clinical conditions.

Finally, the methodological rigor of many DA-based cardiovascular studies is a further strength. The use of matching, regression adjustment, sensitivity analyses, and stratification by demographic variables is consistent across many of the studies reviewed, supporting the internal validity of the results. While causal inference is not possible, the methods employed minimize bias within the limits of observational data.

Despite its considerable strengths, the DA database also has a number of limitations that are inherent to routine clinical data. First, diagnoses depend on physician coding practices, meaning misclassification is possible. For cardiovascular conditions such as HF or AF, diagnostic accuracy may vary based on provider expertise and available diagnostic tools. Some comorbidities, such as NAFLD or early-stage renal dysfunction, may be underdiagnosed, which can influence the observed strength of associations. Moreover, temporal changes in diagnostic coding practices, disease awareness, and clinical guidelines over the 25-year observation period may influence observed associations. Advances in diagnostic technologies and evolving guideline recommendations—particularly for atrial fibrillation, lipid management, and heart failure—may limit comparability between studies conducted in different time periods.

Second, important clinical parameters are inconsistently available in the database. For example, lipid levels, BMI measurements, or imaging results may not be recorded systematically. While laboratory values were available in the LDL-C distance study by Stach et al. [19], laboratory completeness varies widely across the database as a whole. The absence of detailed echocardiographic parameters, imaging data, and biomarkers limits the granularity of cardiovascular phenotyping.

Residual confounding is another major constraint. Lifestyle factors such as smoking, diet, alcohol consumption, and physical activity are not reliably captured in the database. Consequently, even sophisticated matching or regression adjustment cannot eliminate confounding from unmeasured variables. For instance, in the study linking antibiotic use with HF incidence [23], underlying conditions necessitating repeated antibiotic therapy may also contribute to HF risk, complicating interpretation. Future Disease Analyzer–based research may benefit from emerging methodological approaches such as target trial emulation, which explicitly aligns observational analyses with hypothetical randomized trial protocols. Additionally, triangulation with other real-world data sources, registries, or claims databases may help validate observed associations and strengthen causal interpretation.

The database also lacks mortality data, so cardiovascular death cannot be evaluated except through indirect inference from patterns of clinical decline. Additionally, hospital-based diagnoses may not be captured fully unless subsequently documented in outpatient care.

It should also be acknowledged that the selection of the 13 reviewed studies may introduce publication-level selection bias. Although the studies were chosen based on predefined criteria reflecting cardiovascular relevance, methodological rigor, and representative coverage of major cardiovascular domains, other DA-based cardiovascular studies published during the same period were not included. The review, therefore, provides a thematic synthesis rather than a comprehensive systematic evaluation of all DA publications.

Another limitation concerns generalizability beyond Germany. While the DA sample is nationally representative, healthcare systems differ from country to country, so the associations observed in Germany may not apply identically elsewhere. Additionally, since the DA database reflects outpatient care, events that occur only during hospital stays may not be recorded.

Finally, observational DA-based studies cannot establish causality. While several studies reported strong associations—such as NAFLD with HF [22] or obesity with AF incidence [15]—the results should be understood as associations rather than causal effects. This distinction is essential for proper scientific interpretation and for preventing the overstatement of findings.

## 8. Conclusions

The German IQVIA Disease Analyzer database has become a central platform for real-world cardiovascular research. The 13 studies summarized in this review demonstrate their usefulness in examining associations between exposures, comorbidities, and cardiovascular outcomes across millions of patients and multiple clinical domains, including AF, HF, cerebrovascular events, cardiometabolic conditions, lipid management, NAFLD, and emerging issues such as COVID-19-related vascular risks. The database offers major advantages in terms of scale, representativeness, and longitudinal design, enabling high-resolution epidemiological analyses. However, limitations related to coding accuracy, missing clinical details, residual confounding, and the inability to infer causality require careful methodological consideration.

Future research should build on these findings by integrating Disease Analyzer data with complementary real-world sources, applying advanced causal inference methods, and exploring longitudinal changes in cardiovascular care delivery. Such approaches may enhance the translational value of outpatient real-world evidence for cardiovascular prevention and management.

Overall, the evidence summarized in this review primarily contributes to descriptive cardiovascular epidemiology and association research rather than etiological inference. While the Disease Analyzer database enables the identification of clinically meaningful patterns in routine care, causal conclusions cannot be drawn and require cautious interpretation supported by complementary methodological approaches.

## Figures and Tables

**Table 1:** Overview of studies using the Disease Analyzer database for cardiovascular research (2020–2025). Table 1 provides a structured overview of study designs, populations, and main findings to facilitate cross-study comparison and thematic synthesis across cardiovascular domains.

Author, Year [Reference]	Condition Studied	Study Design	Sample Size	Main Findings
Sedighi et al., 2025 [14]	Atrial fibrillation & sex differences in antithrombotic therapy	Retrospective cohort study	166,005	Female patients had lower odds of receiving antithrombotic therapy, and there were sex differences associated with therapy initiation
Sedighi et al., 2025 [15]	Overweight, obesity & incident atrial fibrillation	Retrospective cohort study	400,000	Obesity is strongly associated with a higher incidence of AF
Sedighi et al., 2025 [16]	Sleep disorders & incident heart failure	Retrospective matched cohort study	123,516	Sleep disorders are associated with a higher incidence of HF
Rodemer et al., 2025 [17]	Schizophrenia & cardiovascular diseases	Retrospective cohort study	75,162	Schizophrenia was associated with a higher incidence of cardiovascular disease
Kostev et al., 2025 [18]	Alcoholic & non-alcoholic liver cirrhosis and heart failure	Retrospective cohort study	75,558	Both forms of cirrhosis are associated with higher HF incidence
Stach et al., 2024 [19]	LDL-C goal attainment in high-risk hyperlipidemia	Retrospective claims & EMR analysis	32,963	Most patients failed to reach LDL-C targets, indicating a considerable unmet treatment need
Krieg et al., 2024 [20]	ADHD & dyslipidemia in adults	Retrospective cohort study	32,202	Adults with ADHD showed increased rates of dyslipidemia
Krieg et al., 2022 [21]	Body height & cardiovascular diseases	Retrospective cohort	657,310	Shorter height is associated with a higher incidence of CVD
Roderburg et al., 2023 [22]	NAFLD & risk of new-onset heart failure	Retrospective cohort study	173,966	NAFLD is associated with higher HF incidence independent of comorbidities
Loosen et al., 2023 [23]	Antibiotic use & incident heart failure	Retrospective case–control	162,188	Antibiotic exposure is associated with an increased incidence of HF
Loosen et al., 2023 [24]	Comorbidities of HF patients	Retrospective case–control	324,492	36 diagnoses are more prevalent in HF patients than in controls
Zappacosta et al., 2022 [25]	COVID-19 & vascular events	Retrospective cohort study	58,904	COVID-19 infection is associated with a higher likelihood of subsequent vascular events
Rathmann & Kostev, 2022 [26]	Glucose-lowering drugs & incident stroke/TIA	Retrospective cohort study	312,368	Several glucose-lowering therapies are associated with various stroke risks

## Data Availability

No new data were created or analyzed in this study.

## References

[1] Sherman R.E., Anderson S.A., Dal Pan G.J., Gray G.W., Gross T., Hunter N.L., LaVange L., Marinac-Dabic D., Marks P.W., Robb M.A. (2016). Real-world evidence—What is it and what can it tell us?. N. Engl. J. Med..

[2] Makady A., van Veelen A., Jonsson P., Moseley O., D’Andon A., de Boer A., Hillege H., Klungel O., Goettsch W. (2018). Using real-world data in health technology assessment (HTA) practice: A comparative study of five HTA agencies. Pharmacoeconomics.

[3] Schneeweiss S. (2014). Learning from big health care data. N. Engl. J. Med..

[4] Hernán M.A., Robins J.M. (2016). Using big data to emulate a target trial when a randomized trial is not available. Am. J. Epidemiol..

[5] Benchimol E.I., Smeeth L., Guttmann A., Harron K., Hemkens L.G., Moher D., Petersen I., Sørensen H.T., von Elm E., Langan S.M. (2016). Das RECORD-Statement zum Berichten von Beobachtungsstudien, die routinemäßig gesammelte Gesundheitsdaten verwenden. Z. Evid. Fortbild. Qual. Gesundhwes..

[6] Franklin J.M., Schneeweiss S. (2017). When and how can real-world data analyses substitute for randomized controlled trials?. Clin. Pharmacol. Ther..

[7] Ieva F., Gale C.P., Sharples L.D. (2014). Contemporary roles of registries in clinical cardiology: When do we need randomized trials?. Expert Rev. Cardiovasc. Ther..

[8] Corrigan-Curay J., Sacks L., Woodcock J. (2018). Real-world evidence and real-world data for evaluating drug safety and effectiveness. JAMA.

[9] Garrison L.P., Neumann P.J., Erickson P., Marshall D., Mullins C.D. (2007). Using real-world data for coverage and payment decisions: The ISPOR real-world data task force report. Value Health.

[10] Susukida R., Crum R.M., Ebnesajjad C., Stuart E.A., Mojtabai R. (2017). Generalizability of findings from randomized controlled trials: Application to the national institute of drug abuse clinical trials network. Addiction.

[11] He Z., Tang X., Yang X., Guo Y., George T.J., Charness N., Quan Hem K.B., Hogan W., Bian J. (2020). Clinical trial generalizability assessment in the big data era: A review. Clin. Transl. Sci..

[12] Suvarna V.R. (2018). Real-world evidence (RWE)—Are we ready?. Perspect. Clin. Res..

[13] Averitt A.J., Weng C., Ryan P., Perotte A. (2020). Translating evidence into practice: Eligibility criteria fail to eliminate clinically significant differences between real-world and study populations. NPJ Digit. Med..

[14] Sedighi J., Luedde M., Dinov B., Bengel P., Boettger P., Tanislav C., Sossalla S., Kostev K. (2025). Sex differences in the initiation of antithrombotic therapy in patients with atrial fibrillation in Germany. Clin. Res. Cardiol..

[15] Sedighi J., Luedde M., Boettger P., Bengel P., Bauer P., Sossalla S., Rozo Sánchez S.E., Kostev K. (2025). Overweight, obesity and incident atrial fibrillation: Real-world evidence from 400 000 patients in Germany. Diabetes Obes. Metab..

[16] Sedighi J., Luedde M., Gaensbacher-Kunzendorf J., Hippe H.J., Bauer P., Assmus B., Sossalla S., Kostev K. (2025). Association between sleep disorders and subsequent heart failure. Int. J. Cardiol. Heart Vasc..

[17] Rodemer I., Konrad M., Luedde M., Kostev K. (2025). Association between schizophrenia and cardiovascular diseases: A Retrospective Cohort Study of Primary Care Routine Data in Germany. Brain Sci..

[18] Kostev K., Sedighi J., Sossalla S., Konrad M., Luedde M. (2025). Both Alcoholic and Non-Alcoholic Liver Cirrhosis Are Associated with an Increased Risk of HF—A Cohort Study Including 75,558 Patients. J. Cardiovasc. Dev. Dis..

[19] Stach K., Richter H., Fraass U., Stein A. (2024). Quantifying the ‘distance to LDL-C goal’ in patients at very high cardiovascular risk with hyperlipidaemia in Germany: A retrospective claims database analysis. Ther. Adv. Cardiovasc. Dis..

[20] Krieg S., Konrad M., Krieg A., Kostev K. (2024). What Is the Link between Attention-Deficit/Hyperactivity Disorder (ADHD) and Dyslipidemia in Adults? A German Retro-spective Cohort Study. J. Clin. Med..

[21] Krieg S., Kostev K., Luedde M., Krieg A., Luedde T., Roderburg C., Loosen S.H. (2022). The association between the body height and cardiovascular diseases: A retrospective analysis of 657,310 outpatients in Germany. Eur. J. Med. Res..

[22] Roderburg C., Krieg S., Krieg A., Vaghiri S., Mohr R., Konrad M., Luedde M., Luedde T., Kostev K., Loosen S.H. (2023). Non-Alcoholic Fatty Liver Disease (NAFLD) and risk of new-onset heart failure: A retrospective analysis of 173,966 patients. Clin. Res. Cardiol..

[23] Loosen S.H., Krieg S., Gaensbacher J., Doege C., Krieg A., Luedde T., Luedde M., Roderburg C., Kostev K. (2023). The Association between Antibiotic Use and the Incidence of Heart Failure: A Retrospective Case-Control Study of 162,188 Outpatients. Biomedicines.

[24] Loosen S.H., Roderburg C., Curth O., Gaensbacher J., Joerdens M., Luedde T., Konrad M., Kostev K., Luedde M. (2022). The spectrum of comorbidities at the initial diagnosis of heart failure a case control study. Sci. Rep..

[25] Zappacosta S., Cascarano A., Konrad M., Tanislav C., Kostev K. (2022). Association between COVID-19 and subsequent vascular events in primary care patients in Germany. Public Health.

[26] Rathmann W., Kostev K. (2022). Glucose-lowering drugs and stroke risk. Acta Diabetol..

[27] Doehner W., Boriani G., Potpara T., Blomstrom-Lundqvist C., Passman R., Sposato L.A., Dobrev D., Freedman B., Van Gelder I.C., Glotzer T.V. (2025). Atrial fibrillation burden in clinical practice, research, and technology development: A clinical consensus statement of the European Society of Cardiology Council on Stroke and the European Heart Rhythm Association. Europace.

[28] Westerman S., Wenger N. (2019). Gender Differences in Atrial Fibrillation: A Review of Epidemiology, Management, and Outcomes. Curr. Cardiol. Rev..

[29] Yong C.M., Tremmel J.A., Lansberg M.G., Fan J., Askari M., Turakhia M.P. (2020). Sex Differences in Oral Anticoagulation and Outcomes of Stroke and Intracranial Bleeding in Newly Diagnosed Atrial Fibrillation. J. Am. Heart Assoc..

[30] Teppo K., Jaakkola J., Lehto M., Biancari F., Airaksinen K.E.J. (2021). The impact of mental health conditions on oral anticoagulation therapy and outcomes in patients with atrial fibrillation: A systematic review and meta-analysis. Am. J. Prev. Cardiol..

[31] Frost L., Hune L.J., Vestergaard P. (2005). Overweight and obesity as risk factors for atrial fibrillation or flutter: The Danish Diet, Cancer, and Health Study. Am. J. Med..

[32] Wang T.J., Parise H., Levy D., D’Agostino R.B., Wolf P.A., Vasan R.S., Benjamin E.J. (2004). Obesity and the risk of new-onset atrial fibrillation. JAMA.

[33] Rosenberg M.A., Patton K.K., Sotoodehnia N., Karas M.G., Kizer J.R., Zimetbaum P.J., Chang J.D., Siscovick D., Gottdiener J.S., Kronmal R.A. (2012). The impact of height on the risk of atrial fibrillation: The Cardiovascular Health Study. Eur. Heart J..

[34] Rosenberg M.A., Kaplan R.C., Siscovick D.S., Psaty B.M., Heckbert S.R., Newton-Cheh C., Mukamal K.J. (2014). Genetic Variants Related to Height and Risk of Atrial Fibrillation: The Cardiovascular Health Study. Am. J. Epidemiol..

[35] Nüesch E., Dale C., Palmer T.M., White J., Keating B.J., van Iperen E.P., Goel A., Padmanabhan S., Asselbergs F.W., Verschuren W.M. (2016). Adult height, coronary heart disease and stroke: A multi-locus Mendelian randomization meta-analysis Open Access. Int. J. Epidemiol..

[36] Chen Y., Peng W., Pang M., Zhu B., Liu H., Hu D., Luo Y., Wang S., Wu S., He J. (2024). The effects of psychiatric disorders on the risk of chronic heart failure: A univariable and multivariable Mendelian randomization study. Front. Public Health.

[37] Correll C.U., Solmi M., Veronese N., Bortolato B., Rosson S., Santonastaso P., Thapa-Chhetri N., Fornaro M., Gallicchio D., Collantoni E. (2017). Prevalence, incidence and mortality from cardiovascular disease in patients with pooled and specific severe mental illness: A large-scale meta-analysis of 3,211,768 patients and 113,383,368 controls. World Psychiatry.

[38] Komuro J., Kaneko H., Suzuki Y., Okada A., Fujiu K., Takeda N., Jo T., Morita H., Senoo K., Node K. (2024). Sex Differences in the Relationship Between Schizophrenia and the Development of Cardiovascular Disease. J. Am. Heart Assoc..

[39] Veeneman R.R., Vermeulen J.M., Abdellaoui A., Sanderson E., Wootton R.E., Tadros R., Bezzina C.R., Denys D., Munafò M.R., Verweij K.J.H. (2022). Exploring the Relationship Between Schizophrenia and Cardiovascular Disease: A Genetic Correlation and Multivariable Mendelian Randomization Study Open Access. Schizophr. Bull..

